# Supplementary material for: Association between oral microbiome and seven types of cancers in East Asian population: a two-sample Mendelian randomization analysis
Source: Front Mol Biosci. 2023 Nov 21;10:1327893. doi: 10.3389/fmolb.2023.1327893 (PMC10702768; doi:10.3389/fmolb.2023.1327893)
Supplement: Supplementary file 1 [file DataSheet1.ZIP › Supplementary Materials/MR plots for tongue/tongue═╝/Prostate cancer/pheno.2090_to_prostate cancer_leave_one_out.pdf]

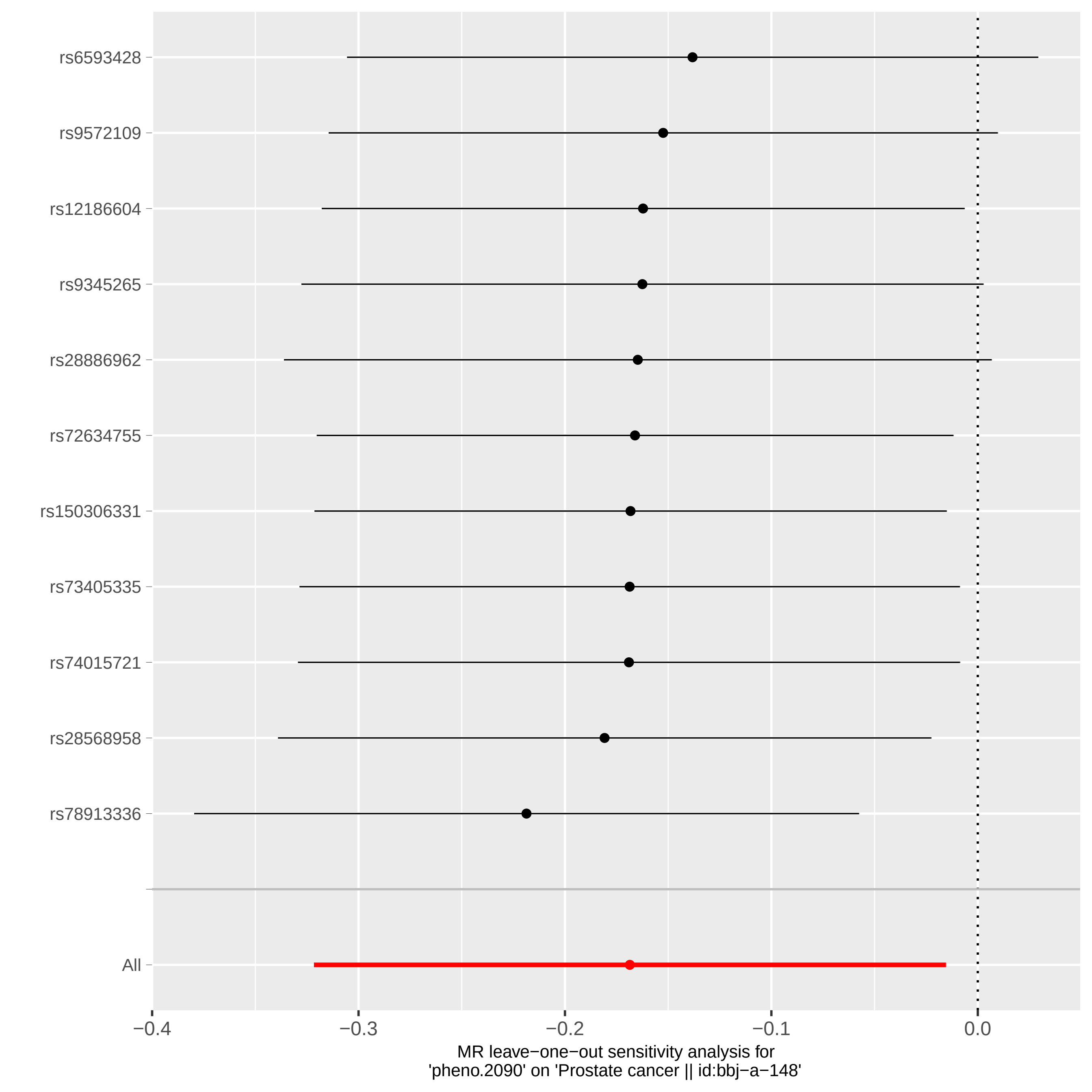

rs6593428
rs9572109
rs12186604
rs9345265
rs28886962
rs72634755
rs150306331
rs73405335
rs74015721
rs28568958
rs78913336
All
−0.4
−0.3
−0.2
−0.1
0.0
MR leave−one−out sensitivity analysis for
'pheno.2090' on 'Prostate cancer || id:bbj−a−148'
